# *catena*-Poly[[diaqua­bis­{μ_2_-3,5-bis­[(pyridin-4-yl)methyl­amino]­benzoato}nickel] monohydrate]

**DOI:** 10.1107/S1600536811038633

**Published:** 2011-09-30

**Authors:** Hai-Wei Kuai, Xiao-Chun Cheng

**Affiliations:** a Faculty of Life Science and Chemical Engineering, Huaiyin Institute of Technology, Huaian 223003, People’s Republic of China

## Abstract

In the title coordination polymer, {[Ni(C_19_H_17_N_4_O_2_)_2_(H_2_O)_2_]·H_2_O}_*n*_, the Ni^2+^ cation is located on an inversion center and coordinated by two carboxyl­ate O atoms from two different 3,5-bis­(pyridin-4-yl­methyl­amino)­benzoate anions, two O atoms from two coordinated water mol­ecules and two N atoms from two different 3,5-bis­(pyridin-4-yl­methyl­amino)­benzoate anions, displaying a slightly distorted NiN_2_O_4_ octa­hedral geometry. Each 3,5-bis­(pyridin-4-yl­methyl­amino)­benzoate anion acts as a μ_2_-bridge, linking different nickel ions into a chain along [010]. In the crystal, adjacent chains are further linked through N—H⋯O, O—H⋯O, O—H⋯N and C—H⋯O hydrogen bonds into a three-dimensional network. The coordinated water mol­ecules and a disordered water mol­ecule of hydration with 0.50 site occupancy play an important role in the formation of these hydrogen-bonding inter­actions.

## Related literature

For background to metal-organic hybrid materials, see: Bradshaw *et al.* (2005 ▶); Das & Bharadwaj (2009 ▶); Hua *et al.* (2010 ▶). For the use of *N*-, or *O*- multidentate donor ligands as building blocks in the construction of infinite frameworks, see: Peng *et al.* (2010 ▶). For related structures, see: Chen *et al.* (2009 ▶); Kuai *et al.* (2011 ▶). 
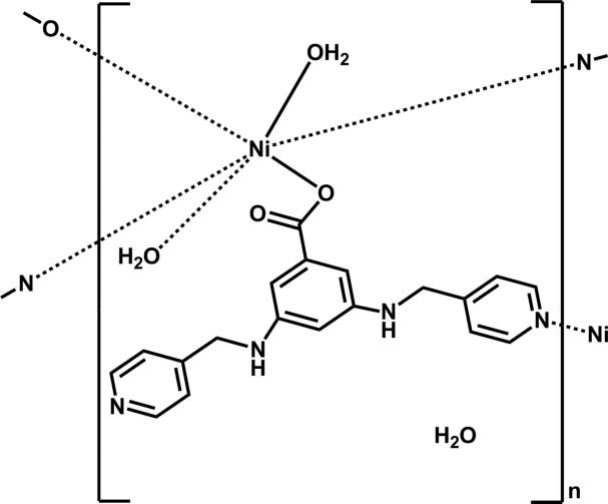


## Experimental

### 

#### Crystal data

[Ni(C_19_H_17_N_4_O_2_)_2_(H_2_O)_2_]·H_2_O
*M* *_r_* = 779.49
Monoclinic, 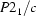

*a* = 10.7786 (10) Å
*b* = 9.3152 (9) Å
*c* = 18.1211 (17) Å
β = 92.324 (1)°
*V* = 1817.9 (3) Å^3^
*Z* = 2
Mo *K*α radiation
μ = 0.60 mm^−1^
*T* = 293 K
0.22 × 0.20 × 0.18 mm

#### Data collection

Bruker APEXII CCD area-detector diffractometer
Absorption correction: multi-scan (*SADABS*; Sheldrick, 1996 ▶) *T*_min_ = 0.880, *T*_max_ = 0.900
11129 measured reflections
4300 independent reflections
2688 reflections with *I* > 2σ(*I*)
*R*_int_ = 0.061

#### Refinement

*R*[*F* ^2^ > 2σ(*F* ^2^)] = 0.043
*wR*(*F* ^2^) = 0.094
*S* = 0.90
4300 reflections
250 parameters
H-atom parameters constrained
Δρ_max_ = 0.44 e Å^−3^
Δρ_min_ = −0.47 e Å^−3^

### 

Data collection: *APEX2* (Bruker, 2008 ▶); cell refinement: *SAINT* (Bruker, 2008 ▶); data reduction: *SAINT*; program(s) used to solve structure: *SHELXS97* (Sheldrick, 2008 ▶); program(s) used to refine structure: *SHELXL97* (Sheldrick, 2008 ▶); molecular graphics: *DIAMOND* (Brandenburg, 2000 ▶); software used to prepare material for publication: *SHELXTL* (Sheldrick, 2008 ▶).

## Supplementary Material

Crystal structure: contains datablock(s) I, Global. DOI: 10.1107/S1600536811038633/pv2448sup1.cif

Structure factors: contains datablock(s) I. DOI: 10.1107/S1600536811038633/pv2448Isup2.hkl

Supplementary material file. DOI: 10.1107/S1600536811038633/pv2448Isup4.cdx

Additional supplementary materials:  crystallographic information; 3D view; checkCIF report

## Figures and Tables

**Table 1:** Hydrogen-bond geometry (Å, °)

*D*—H⋯*A*	*D*—H	H⋯*A*	*D*⋯*A*	*D*—H⋯*A*
N12—H10⋯O3^i^	0.92	2.13	3.023 (2)	163
O3—H18⋯O2	0.86	1.77	2.627 (2)	169
O4—H21⋯O2^ii^	0.90	2.09	2.701 (5)	125
O4—H20⋯O2^iii^	1.06	1.72	2.690 (5)	150
O3—H19⋯N31^iv^	0.97	1.87	2.787 (3)	156
C2—H1⋯O4^v^	0.93	2.58	3.505 (6)	172
C37—H16⋯O4^v^	0.97	2.47	3.440 (8)	176

Symmetry codes: (i) 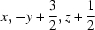; (ii) 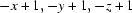; (iii) 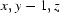; (iv) 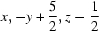; (v) 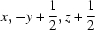.

**Fig. 1.**
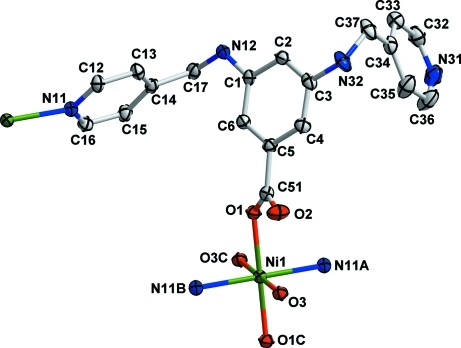
The coordination environment of nickel ion in the title complex with the ellipsoids drawn at the 30% probability level; hydrogen atoms have been omitted for clarity. Symmetry codes: (A) x, 1 + y, z; (B) -x, 1 - y, 1 - z; (C) -x, 2 - y, 1 - z.

**Fig. 2.**
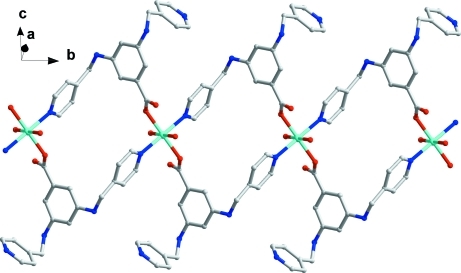
A one-dimensional chain formed from nickel ions and 3,5-bis(pyridin-4-ylmethylamino)benzoate anions.

**Fig. 3.**
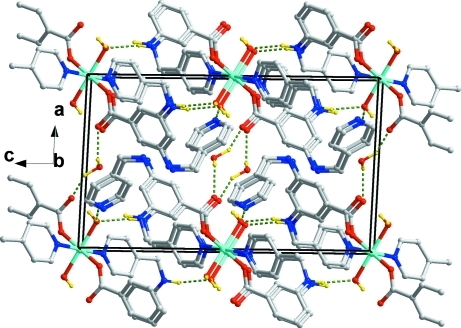
Unit cell packing of the title complex showing the three-dimensional network constructed from one-dimensional chains via hydrogen bonding.

## supplementary crystallographic information

### Comment

During the past few decades, growing interests have been focused on the rapidly expanding field of crystal engineering of metal-organic frameworks (MOFs) due to their intriguing architectures as well as their tremendous potential applications in heterogeneous catalysis, ion-recognition, nonlinear optics and molecular adsorption (Bradshaw *et al.*, 2005; Das & Bharadwaj, 2009; Hua *et al.*, 2010). One of the effective strategies for construction of such polymers is to select suitable multidentate organic ligands as building blocks to link metal centers into infinite framework. Among popularly employed organic ligands, N–, or O– multidentate donor ligands are regarded as excellent candidates for building the blocks of desirable frameworks (Peng *et al.*, 2010). Herein, we report the crystal structure of the title coordination polymer.

The asymmetric unit of the title complex consists of half of a nickel ion, a 3,5-bis(pyridin-4-ylmethylamino)benzoate anion, a coordinated water molecule, and one half water molecule of crystallization. The Ni ion is located on an inversion center and coordinated by two carboxylate O atoms from two different 3,5-bis(pyridin-4-ylmethylamino)benzoate anions, two O atoms from two coordinated water molecules, and two N atoms from two different 3,5-bis(pyridin-4-ylmethylamino)benzoate anions, displaying a slightly distorted NiN_2_O_4_ octahedral geometry. (Fig. 1). Each 3,5-bis(pyridin-4-ylmethylamino)benzoate anion acts as a µ_2_-bridge, linking different nickel ions to form a one-dimensional chain (Fig. 2). In the crystal structure, adjacent chains are further linked through N—H···O, O—H···O, O—H···N and C—H···O hydrogen bonds into a three-dimensional network (Fig. 3 and Table 1). Water molecules as donor or acceptor, including coordinated water molecules and lattice water molecule, play very important roles in the formation of these hydrogen bonding interactions.

### Experimental

A mixture of nickel nitrate hexahydrate (29.1 mg, 0.1 mmol), 3,5-bis(pyridin-4-ylmethylamino)benzoic acid (33.4 mg, 0.1 mmol), and potassium hydroxide (5.61 mg, 0.1 mmol) in 8 ml H_2_O was sealed in a 16 ml Teflon-lined stainless steel container and heated to 373 K for 3 days. After cooling to the room temperature, green block crystals of the title complex were obtained.

### Refinement

The hydrogen atoms bonded to C atoms were included in geometrically idealized positions and constrained to ride on their parent atoms, with C—H = 0.93–0.97 Å and *U*_iso_(H) = 1.2*U*_eq_(C). The hydrogen atoms bonded to N and O atoms were located from the difference Fourier maps and fixed at those positions with *U*_iso_(H) = 1.2*U*_eq_(N or O)].

### Crystal data

**Table:**

[Ni(C_19_H_17_N_4_O_2_)_2_(H_2_O)_2_]·H_2_O	*F*(000) = 816
*M**_r_* = 779.49	*D*_x_ = 1.424 Mg m^−^^3^
Monoclinic, *P*2_1_/*c*	Mo *K*α radiation, λ = 0.71073 Å
Hall symbol: -P 2ybc	Cell parameters from 2160 reflections
*a* = 10.7786 (10) Å	θ = 2.5–22.6°
*b* = 9.3152 (9) Å	µ = 0.60 mm^−^^1^
*c* = 18.1211 (17) Å	*T* = 293 K
β = 92.324 (1)°	Block, green
*V* = 1817.9 (3) Å^3^	0.22 × 0.20 × 0.18 mm
*Z* = 2	

### Data collection

**Table:**

Bruker APEXII CCD area-detector diffractometer	4300 independent reflections
Radiation source: fine-focus sealed tube	2688 reflections with *I* > 2σ(*I*)
graphite	*R*_int_ = 0.061
φ and ω scans	θ_max_ = 28.0°, θ_min_ = 1.9°
Absorption correction: multi-scan (SADBAS; Sheldrick, 1996)	*h* = −14→11
*T*_min_ = 0.880, *T*_max_ = 0.900	*k* = −12→11
11129 measured reflections	*l* = −23→23

### Refinement

**Table:**

Refinement on *F*^2^	Primary atom site location: structure-invariant direct methods
Least-squares matrix: full	Secondary atom site location: difference Fourier map
*R*[*F*^2^ > 2σ(*F*^2^)] = 0.043	Hydrogen site location: inferred from neighbouring sites
*wR*(*F*^2^) = 0.094	H-atom parameters constrained
*S* = 0.90	*w* = 1/[σ^2^(*F*_o_^2^) + (0.0321*P*)^2^] where *P* = (*F*_o_^2^ + 2*F*_c_^2^)/3
4300 reflections	(Δ/σ)_max_ < 0.001
250 parameters	Δρ_max_ = 0.44 e Å^−^^3^
0 restraints	Δρ_min_ = −0.47 e Å^−^^3^

### Special details

**Table:**

Geometry. All e.s.d.'s (except the e.s.d. in the dihedral angle between two l.s. planes) are estimated using the full covariance matrix. The cell e.s.d.'s are taken into account individually in the estimation of e.s.d.'s in distances, angles and torsion angles; correlations between e.s.d.'s in cell parameters are only used when they are defined by crystal symmetry. An approximate (isotropic) treatment of cell e.s.d.'s is used for estimating e.s.d.'s involving l.s. planes.
Refinement. Refinement of *F*^2^ against ALL reflections. The weighted *R*-factor *wR* and goodness of fit *S* are based on *F*^2^, conventional *R*-factors *R* are based on *F*, with *F* set to zero for negative *F*^2^. The threshold expression of *F*^2^ > σ(*F*^2^) is used only for calculating *R*-factors(gt) *etc*. and is not relevant to the choice of reflections for refinement. *R*-factors based on *F*^2^ are statistically about twice as large as those based on *F*, and *R*- factors based on ALL data will be even larger.

### Fractional atomic coordinates and isotropic or equivalent isotropic displacement parameters (Å^2^)

**Table:**

	*x*	*y*	*z*	*U*_iso_*/*U*_eq_	Occ. (<1)
C1	0.2314 (2)	0.6148 (2)	0.75121 (13)	0.0339 (6)	
C2	0.3407 (2)	0.6653 (3)	0.78657 (13)	0.0378 (6)	
H1	0.3675	0.6260	0.8316	0.045*	
C3	0.4095 (2)	0.7733 (3)	0.75505 (13)	0.0377 (6)	
C4	0.3705 (2)	0.8302 (3)	0.68700 (13)	0.0394 (6)	
H2	0.4173	0.9009	0.6650	0.047*	
C5	0.2619 (2)	0.7814 (2)	0.65213 (12)	0.0324 (5)	
C6	0.1920 (2)	0.6747 (2)	0.68380 (12)	0.0327 (5)	
H3	0.1189	0.6430	0.6601	0.039*	
C12	0.1299 (2)	0.1913 (2)	0.61577 (12)	0.0344 (6)	
H4	0.1999	0.1400	0.6032	0.041*	
C13	0.1411 (2)	0.2878 (2)	0.67343 (12)	0.0359 (6)	
H5	0.2170	0.2994	0.6990	0.043*	
C14	0.0392 (2)	0.3673 (2)	0.69312 (12)	0.0323 (5)	
C15	−0.0692 (2)	0.3470 (2)	0.65150 (13)	0.0390 (6)	
H6	−0.1396	0.4001	0.6616	0.047*	
C16	−0.0735 (2)	0.2485 (3)	0.59503 (13)	0.0389 (6)	
H7	−0.1479	0.2371	0.5678	0.047*	
C17	0.0430 (2)	0.4665 (2)	0.75874 (13)	0.0389 (6)	
H8	−0.0025	0.5533	0.7456	0.047*	
H9	0.0009	0.4206	0.7987	0.047*	
C32	0.2739 (3)	1.0393 (3)	0.97764 (16)	0.0556 (8)	
H11	0.2331	1.0256	1.0213	0.067*	
C33	0.3599 (2)	0.9375 (3)	0.95793 (15)	0.0499 (7)	
H12	0.3756	0.8582	0.9880	0.060*	
C34	0.4219 (2)	0.9538 (3)	0.89414 (15)	0.0429 (6)	
C35	0.3910 (3)	1.0717 (3)	0.85121 (17)	0.0646 (9)	
H13	0.4277	1.0856	0.8062	0.078*	
C36	0.3051 (3)	1.1692 (3)	0.87565 (19)	0.0748 (10)	
H14	0.2877	1.2495	0.8466	0.090*	
C37	0.5200 (2)	0.8495 (3)	0.87000 (15)	0.0549 (8)	
H15	0.6009	0.8861	0.8861	0.066*	
H16	0.5081	0.7587	0.8949	0.066*	
C51	0.2157 (2)	0.8553 (2)	0.58246 (13)	0.0325 (5)	
N11	0.02360 (17)	0.16775 (19)	0.57703 (10)	0.0312 (4)	
N12	0.16700 (19)	0.5057 (2)	0.78483 (10)	0.0402 (5)	
H10	0.1781	0.4972	0.8355	0.048*	
N31	0.2462 (2)	1.1551 (3)	0.93783 (14)	0.0618 (7)	
N32	0.52039 (17)	0.8233 (2)	0.79128 (12)	0.0503 (6)	
H17	0.5447	0.8995	0.7696	0.060*	
Ni1	0.0000	1.0000	0.5000	0.02782 (13)	
O1	0.09988 (14)	0.86368 (16)	0.57090 (8)	0.0344 (4)	
O2	0.29410 (15)	0.9082 (2)	0.54080 (10)	0.0502 (5)	
O3	0.15872 (13)	1.06418 (16)	0.44745 (8)	0.0342 (4)	
H18	0.2116	1.0178	0.4756	0.041*	
H19	0.1664	1.1683	0.4476	0.041*	
O4	0.4743 (5)	−0.0193 (9)	0.4494 (3)	0.204 (4)	0.50
H20	0.4269	−0.0406	0.4985	0.245*	0.50
H21	0.5132	0.0652	0.4551	0.245*	0.50

### Atomic displacement parameters (Å^2^)

**Table:**

	*U*^11^	*U*^22^	*U*^33^	*U*^12^	*U*^13^	*U*^23^
C1	0.0435 (14)	0.0271 (13)	0.0308 (13)	0.0031 (11)	−0.0003 (11)	−0.0066 (10)
C2	0.0454 (15)	0.0369 (15)	0.0302 (14)	0.0113 (12)	−0.0078 (11)	−0.0043 (11)
C3	0.0293 (13)	0.0405 (15)	0.0427 (15)	0.0071 (11)	−0.0058 (11)	−0.0113 (12)
C4	0.0347 (14)	0.0393 (15)	0.0441 (16)	0.0011 (11)	−0.0009 (12)	−0.0003 (12)
C5	0.0333 (13)	0.0313 (13)	0.0323 (13)	0.0051 (10)	−0.0006 (10)	−0.0001 (10)
C6	0.0356 (13)	0.0312 (13)	0.0310 (13)	0.0009 (10)	−0.0038 (10)	−0.0028 (10)
C12	0.0332 (13)	0.0323 (14)	0.0380 (14)	−0.0011 (11)	0.0047 (11)	−0.0062 (11)
C13	0.0360 (14)	0.0371 (14)	0.0345 (14)	−0.0082 (11)	0.0002 (11)	−0.0060 (11)
C14	0.0426 (14)	0.0250 (13)	0.0297 (13)	−0.0068 (11)	0.0069 (11)	0.0010 (10)
C15	0.0398 (14)	0.0313 (14)	0.0458 (16)	0.0050 (11)	0.0023 (12)	−0.0061 (11)
C16	0.0354 (14)	0.0368 (15)	0.0439 (16)	0.0017 (11)	−0.0064 (12)	−0.0030 (11)
C17	0.0548 (16)	0.0313 (15)	0.0313 (14)	−0.0065 (11)	0.0106 (12)	−0.0029 (10)
C32	0.0563 (19)	0.064 (2)	0.0470 (18)	−0.0080 (15)	0.0080 (14)	−0.0100 (15)
C33	0.0539 (18)	0.0496 (17)	0.0457 (17)	−0.0045 (14)	−0.0064 (14)	−0.0002 (14)
C34	0.0396 (15)	0.0410 (16)	0.0476 (17)	−0.0071 (12)	−0.0042 (13)	−0.0096 (13)
C35	0.085 (2)	0.0425 (18)	0.069 (2)	−0.0043 (17)	0.0315 (18)	−0.0008 (16)
C36	0.108 (3)	0.0388 (18)	0.079 (2)	0.0121 (18)	0.028 (2)	0.0042 (16)
C37	0.0401 (16)	0.069 (2)	0.0540 (19)	0.0042 (14)	−0.0149 (13)	−0.0180 (15)
C51	0.0373 (14)	0.0279 (13)	0.0321 (14)	0.0001 (11)	−0.0007 (11)	−0.0008 (10)
N11	0.0333 (11)	0.0293 (11)	0.0310 (11)	−0.0016 (9)	−0.0002 (8)	−0.0021 (8)
N12	0.0643 (14)	0.0322 (11)	0.0237 (10)	−0.0067 (11)	−0.0039 (9)	−0.0009 (9)
N31	0.0758 (18)	0.0476 (16)	0.0629 (17)	0.0067 (13)	0.0152 (14)	−0.0085 (13)
N32	0.0350 (12)	0.0637 (16)	0.0515 (14)	0.0028 (11)	−0.0086 (10)	−0.0131 (12)
Ni1	0.0319 (2)	0.0265 (2)	0.0248 (2)	−0.00238 (19)	−0.00169 (16)	−0.00021 (18)
O1	0.0327 (9)	0.0358 (10)	0.0340 (9)	−0.0021 (7)	−0.0056 (7)	0.0060 (7)
O2	0.0358 (10)	0.0643 (13)	0.0509 (12)	−0.0005 (9)	0.0071 (8)	0.0203 (10)
O3	0.0401 (10)	0.0308 (9)	0.0313 (9)	−0.0035 (7)	−0.0020 (7)	0.0042 (7)
O4	0.126 (5)	0.383 (11)	0.105 (5)	−0.158 (6)	0.030 (4)	−0.026 (6)

### Geometric parameters (Å, °)

**Table:**

C1—N12	1.386 (3)	C32—H11	0.9300
C1—C6	1.393 (3)	C33—C34	1.367 (3)
C1—C2	1.400 (3)	C33—H12	0.9300
C2—C3	1.387 (3)	C34—C35	1.379 (4)
C2—H1	0.9300	C34—C37	1.514 (3)
C3—C4	1.392 (3)	C35—C36	1.383 (4)
C3—N32	1.418 (3)	C35—H13	0.9300
C4—C5	1.383 (3)	C36—N31	1.322 (3)
C4—H2	0.9300	C36—H14	0.9300
C5—C6	1.386 (3)	C37—N32	1.447 (3)
C5—C51	1.505 (3)	C37—H15	0.9700
C6—H3	0.9300	C37—H16	0.9700
C12—N11	1.337 (3)	C51—O2	1.256 (3)
C12—C13	1.380 (3)	C51—O1	1.260 (3)
C12—H4	0.9300	N11—Ni1^i^	2.1038 (18)
C13—C14	1.383 (3)	N12—H10	0.9247
C13—H5	0.9300	N32—H17	0.8583
C14—C15	1.378 (3)	Ni1—O1^ii^	2.0761 (15)
C14—C17	1.505 (3)	Ni1—O1	2.0761 (15)
C15—C16	1.374 (3)	Ni1—O3	2.0792 (14)
C15—H6	0.9300	Ni1—O3^ii^	2.0792 (14)
C16—N11	1.340 (3)	Ni1—N11^iii^	2.1038 (18)
C16—H7	0.9300	Ni1—N11^iv^	2.1038 (18)
C17—N12	1.446 (3)	O3—H18	0.8647
C17—H8	0.9700	O3—H19	0.9729
C17—H9	0.9700	O4—H20	1.0633
C32—N31	1.325 (3)	O4—H21	0.8961
C32—C33	1.383 (4)		
			
N12—C1—C6	122.6 (2)	C35—C34—C37	120.2 (3)
N12—C1—C2	118.2 (2)	C34—C35—C36	119.5 (3)
C6—C1—C2	119.2 (2)	C34—C35—H13	120.3
C3—C2—C1	120.6 (2)	C36—C35—H13	120.3
C3—C2—H1	119.7	N31—C36—C35	124.0 (3)
C1—C2—H1	119.7	N31—C36—H14	118.0
C2—C3—C4	119.6 (2)	C35—C36—H14	118.0
C2—C3—N32	120.1 (2)	N32—C37—C34	115.0 (2)
C4—C3—N32	120.3 (2)	N32—C37—H15	108.5
C5—C4—C3	120.0 (2)	C34—C37—H15	108.5
C5—C4—H2	120.0	N32—C37—H16	108.5
C3—C4—H2	120.0	C34—C37—H16	108.5
C4—C5—C6	120.7 (2)	H15—C37—H16	107.5
C4—C5—C51	118.5 (2)	O2—C51—O1	124.2 (2)
C6—C5—C51	120.6 (2)	O2—C51—C5	118.4 (2)
C5—C6—C1	119.9 (2)	O1—C51—C5	117.4 (2)
C5—C6—H3	120.1	C12—N11—C16	116.2 (2)
C1—C6—H3	120.1	C12—N11—Ni1^i^	123.26 (15)
N11—C12—C13	123.5 (2)	C16—N11—Ni1^i^	120.24 (15)
N11—C12—H4	118.3	C1—N12—C17	120.95 (19)
C13—C12—H4	118.3	C1—N12—H10	116.9
C12—C13—C14	119.8 (2)	C17—N12—H10	112.6
C12—C13—H5	120.1	C36—N31—C32	116.0 (3)
C14—C13—H5	120.1	C3—N32—C37	118.4 (2)
C15—C14—C13	116.8 (2)	C3—N32—H17	109.1
C15—C14—C17	120.8 (2)	C37—N32—H17	109.0
C13—C14—C17	122.4 (2)	O1^ii^—Ni1—O1	180.00 (7)
C16—C15—C14	120.1 (2)	O1^ii^—Ni1—O3	87.50 (6)
C16—C15—H6	120.0	O1—Ni1—O3	92.50 (6)
C14—C15—H6	120.0	O1^ii^—Ni1—O3^ii^	92.50 (6)
N11—C16—C15	123.6 (2)	O1—Ni1—O3^ii^	87.50 (6)
N11—C16—H7	118.2	O3—Ni1—O3^ii^	180.0
C15—C16—H7	118.2	O1^ii^—Ni1—N11^iii^	89.89 (7)
N12—C17—C14	114.09 (19)	O1—Ni1—N11^iii^	90.11 (7)
N12—C17—H8	108.7	O3—Ni1—N11^iii^	89.39 (6)
C14—C17—H8	108.7	O3^ii^—Ni1—N11^iii^	90.61 (6)
N12—C17—H9	108.7	O1^ii^—Ni1—N11^iv^	90.11 (7)
C14—C17—H9	108.7	O1—Ni1—N11^iv^	89.89 (7)
H8—C17—H9	107.6	O3—Ni1—N11^iv^	90.61 (6)
N31—C32—C33	123.8 (3)	O3^ii^—Ni1—N11^iv^	89.39 (6)
N31—C32—H11	118.1	N11^iii^—Ni1—N11^iv^	180.00 (7)
C33—C32—H11	118.1	C51—O1—Ni1	128.76 (15)
C34—C33—C32	119.9 (3)	Ni1—O3—H18	96.9
C34—C33—H12	120.1	Ni1—O3—H19	110.9
C32—C33—H12	120.1	H18—O3—H19	116.3
C33—C34—C35	116.8 (3)	H20—O4—H21	107.8
C33—C34—C37	123.0 (3)		
			
N12—C1—C2—C3	179.4 (2)	C33—C34—C37—N32	142.8 (3)
C6—C1—C2—C3	−0.1 (3)	C35—C34—C37—N32	−37.1 (4)
C1—C2—C3—C4	−1.1 (3)	C4—C5—C51—O2	31.6 (3)
C1—C2—C3—N32	−179.8 (2)	C6—C5—C51—O2	−153.6 (2)
C2—C3—C4—C5	1.6 (4)	C4—C5—C51—O1	−146.4 (2)
N32—C3—C4—C5	−179.7 (2)	C6—C5—C51—O1	28.4 (3)
C3—C4—C5—C6	−0.9 (4)	C13—C12—N11—C16	2.7 (3)
C3—C4—C5—C51	174.0 (2)	C13—C12—N11—Ni1^i^	−170.66 (17)
C4—C5—C6—C1	−0.4 (3)	C15—C16—N11—C12	−2.4 (4)
C51—C5—C6—C1	−175.1 (2)	C15—C16—N11—Ni1^i^	171.21 (18)
N12—C1—C6—C5	−178.6 (2)	C6—C1—N12—C17	−11.8 (3)
C2—C1—C6—C5	0.9 (3)	C2—C1—N12—C17	168.7 (2)
N11—C12—C13—C14	−0.8 (4)	C14—C17—N12—C1	82.0 (3)
C12—C13—C14—C15	−1.6 (3)	C35—C36—N31—C32	0.1 (5)
C12—C13—C14—C17	175.6 (2)	C33—C32—N31—C36	0.9 (4)
C13—C14—C15—C16	2.0 (3)	C2—C3—N32—C37	−44.2 (3)
C17—C14—C15—C16	−175.3 (2)	C4—C3—N32—C37	137.1 (3)
C14—C15—C16—N11	0.1 (4)	C34—C37—N32—C3	−56.3 (3)
C15—C14—C17—N12	−164.7 (2)	O2—C51—O1—Ni1	−16.3 (3)
C13—C14—C17—N12	18.1 (3)	C5—C51—O1—Ni1	161.54 (15)
N31—C32—C33—C34	0.0 (4)	O1^ii^—Ni1—O1—C51	−87 (100)
C32—C33—C34—C35	−1.9 (4)	O3—Ni1—O1—C51	12.11 (19)
C32—C33—C34—C37	178.1 (2)	O3^ii^—Ni1—O1—C51	−167.89 (19)
C33—C34—C35—C36	2.9 (4)	N11^iii^—Ni1—O1—C51	101.51 (19)
C37—C34—C35—C36	−177.2 (3)	N11^iv^—Ni1—O1—C51	−78.49 (19)
C34—C35—C36—N31	−2.0 (5)		

Symmetry codes: (i) *x*, *y*−1, *z*; (ii) −*x*, −*y*+2, −*z*+1; (iii) −*x*, −*y*+1, −*z*+1; (iv) *x*, *y*+1, *z*.

### Hydrogen-bond geometry (Å, °)

**Table:**

*D*—H···*A*	*D*—H	H···*A*	*D*···*A*	*D*—H···*A*
N12—H10···O3^v^	0.92	2.13	3.023 (2)	163.
O3—H18···O2	0.86	1.77	2.627 (2)	169.
O4—H21···O2^vi^	0.90	2.09	2.701 (5)	125.
O4—H20···O2^i^	1.06	1.72	2.690 (5)	150.
O3—H19···N31^vii^	0.97	1.87	2.787 (3)	156.
C2—H1···O4^viii^	0.93	2.58	3.505 (6)	172.
C37—H16···O4^viii^	0.97	2.47	3.440 (8)	176.

Symmetry codes: (v) *x*, −*y*+3/2, *z*+1/2; (vi) −*x*+1, −*y*+1, −*z*+1; (i) *x*, *y*−1, *z*; (vii) *x*, −*y*+5/2, *z*−1/2; (viii) *x*, −*y*+1/2, *z*+1/2.

## References

[bb1] Bradshaw, D., Claridge, J. B., Cussen, E. J., Prior, T. J. & Rosseinsky, M. J. (2005). *Acc. Chem. Res.* **38**, 273–282. 10.1021/ar0401606 15835874

[bb2] Brandenburg, K. (2000). *DIAMOND* Crystal Impact GbR, Bonn, Germany.

[bb3] Bruker (2008). *APEX2* and *SAINT* Bruker AXS Inc., Madison, Wisconsin, USA.

[bb4] Chen, M.-S., Chen, S.-S., Okamura, T.-A., Su, Z., Sun, W.-Y. & Ueyama, N. (2009). *J. Coord. Chem.* **62**, 2421–2428.

[bb5] Das, M. C. & Bharadwaj, P. K. (2009). *J. Am. Chem. Soc.* **131**, 10942–10943. 10.1021/ja9006035 19621875

[bb6] Hua, Q., Zhao, Y., Xu, G.-C., Chen, M.-S., Su, Z., Cai, K. & Sun, W.-Y. (2010). *Cryst. Growth Des.* **10**, 2553–2562.

[bb7] Kuai, H.-W., Cheng, X.-C. & Zhu, X.-H. (2011). *J. Coord. Chem.* **64**, 1636–1644.

[bb8] Peng, G., Qiu, Y.-C., Liu, Z.-H., Liu, B. & Deng, H. (2010). *Cryst. Growth Des.* **10**, 114–121.

[bb9] Sheldrick, G. M. (1996). *SADABS* University of Göttingen, Germany.

[bb10] Sheldrick, G. M. (2008). *Acta Cryst.* A**64**, 112–122. 10.1107/S0108767307043930 18156677

